# Supplementary material for: The Risk of Psychological Stress on Cancer Recurrence: A Systematic Review
Source: Cancers (Basel). 2021 Nov 19;13(22):5816. doi: 10.3390/cancers13225816 (PMC8616395; doi:10.3390/cancers13225816)
Supplement: Supplementary file 1 [file cancers-13-05816-s001.zip › cancers-1449172 supple for XML.pdf]

# Supplementary Materials: The Risk of Psychological Stress on Cancer Recurrence: A Systematic Review

**Hyeon-Muk Oh and Chang-Gue Son**

**Supplementary Table S1.** Newcastle-Ottawa scale for observational studies and Risk of Bias tool (RoB 2.0) for RCTs.

**Newcastle-Ottawa scale for observational studies**

| Study | Selection | | | | Comparability | Outcome | | | Total |
|---|---|---|---|---|---|---|---|---|---|
| | A | B | C | D | E | F | G | H | |
| Groenvold et al. (2007) [28] | * | * | * | - | ** | * | * | * | 7 |
| Saquib et al. (2011) [29] | * | * | - | - | ** | * | * | * | 6 |
| Olsen et al. (2012) [30] | * | * | * | - | ** | * | * | * | 7 |
| Liu et al. (2016) [31] | * | * | - | - | ** | * | * | * | 6 |

[a]A: Representativeness of the exposed cohort; B: Selection of the nonexposed cohort; C: Ascertainment of cohort; D: Demonstration that outcome of interest not present at the start of the study; E: Comparability of cohort; F: Assessment of outcome; G: Follow-upped long enough until outcomes occur.

[b]The stars (*) are awarded for meeting each criterion. A maximum of one star for item within the Selection and Outcome categories and maximum of two stars can be given for Comparability.

**Risk of bias tool (RoB 2.0) for RCTs**

| Study | Bias arising from the randomization process | Bias due to deviations from intended interventions | Bias due to missing outcome data | Bias in measurement of the outcome | Bias in selection of the reported result |
|---|---|---|---|---|---|
| Andersen et al. (2008) [32] | Some concerns | Low | Low | Low | Low |
| Stagl et al. (2015) [33] | Some concerns | Low | Low | Low | Low |
